# Draft Genome Sequence of a Canine Isolate of Methicillin-Resistant *Staphylococcus haemolyticus*

**DOI:** 10.1128/genomeA.00146-17

**Published:** 2017-04-06

**Authors:** David C. Bean, Sarah M. Wigmore, David W. Wareham

**Affiliations:** a Faculty of Science & Technology, Federation University Australia, Ballarat, Victoria, Australia; b Antimicrobial Research Group, Blizard Institute, Barts and The London School of Medicine and Dentistry, Queen Mary University London, London, United Kingdom

## Abstract

*Staphylococcus haemolyticus* strain SW007 was isolated from a nasal swab taken from a healthy dog. The isolate is resistant to methicillin, mupirocin, macrolides, and sulfonamides. The SW007 draft genome is 2,325,410 bp and contains 2,277 coding sequences, including 60 tRNAs and nine complete rRNA-coding regions.

## GENOME ANNOUNCEMENT

Coagulase-negative staphylococci (CNS) have been considered reservoirs of antibiotic resistance genes with potential for transmission to pathogenic staphylococci, with *Staphylococcus haemolyticus* being particularly notorious for the acquisition of drug resistance (1). Methicillin-resistant *S. haemolyticus* has previously been documented as a cause of infection in both humans (2) and companion animals, including dogs (3). To better understand this potential resistance gene pool in companion animals, we sequenced a multidrug-resistant isolate of *S. haemolyticus* recovered from a nasal swab taken from a 4-year-old neutered female golden retriever living in Ballarat, Australia.

The isolate was recovered from a nasal swab after enrichment in tryptic soya broth supplemented with 8% NaCl and subsequent subculture onto Baird-Parker agar. The isolate was coagulase negative in rabbit plasma, DNase negative, β-galactosidase negative, urease negative, and novobiocin sensitive. Analysis of the 16S rRNA and the housekeeping genes *dnaJ* (4), *rpoB* (5), and *tuf* (6) all identified the isolate as* S. haemolyticus* at ≥99% sequence identity.

Sequencing was performed on the Illumina MiSeq platform (Illumina, Inc., San Diego, CA), and the closest reference genome was identified using Kraken. The reads were mapped to the reference genome using the Burrows-Wheeler aligner “mem” (BWA-mem) algorithm version 2. A *de novo* assembly of the reads was performed using SPAdes version 3.7.1, and the reads were again mapped back to the resultant contigs using BWA-mem. The assembly comprised 27 contigs (>1,000 bp), with a total length of 2,325,410 bp, a G+C content of 32.59%, and an *N*_50_ value of 172,773 bp. Annotation was performed by the NCBI Prokaryote Genome Annotation Pipeline (PGAP), which showed a total of 2,277 coding sequences, including at least 60 tRNAs and nine complete rRNAs.

In addition to *blaZ-* and *mecA*-mediated resistance to β-lactams, the isolate was resistant to three other classes of drugs: mupirocin resistance was imparted by the *mupA* gene, which encodes a mupirocin-resistant isoleucine-tRNA ligase; macrolide resistance (erythromycin, clarithromycin, azithromycin, but not clindamycin) was imparted by the *mph*(C) and *msr*(A) gene pair; and sulfonamide resistance presumably was imparted by mutations in the *folP* gene, encoding dihydropteroate synthase. Indeed, the predicted amino acid sequence contained substitutions at seven sites (two of which are novel, T31K and L64I) previously identified in sulfonamide-resistant isolates of *Staphylococcus aureus* (7).

### Accession number(s).

This whole-genome shotgun project has been deposited at DDBJ/ENA/GenBank under the accession no. MTIZ00000000. The version described in this paper is version MTIZ01000000.
